# ERα and ERβ Homodimers in the Same Cellular Context Regulate Distinct Transcriptomes and Functions

**DOI:** 10.3389/fendo.2022.930227

**Published:** 2022-07-06

**Authors:** Dandan Song, Huan He, Rajitha Indukuri, Zhiqiang Huang, Lina Stepanauskaite, Indranil Sinha, Lars-Arne Haldosén, Chunyan Zhao, Cecilia Williams

**Affiliations:** ^1^ Clinical Medical Research Center for Women and Children Diseases, Maternal and Child Health Care Hospital of Shandong Province, Jinan, China; ^2^ Department of Biosciences and Nutrition, Karolinska Institutet, Huddinge, Sweden; ^3^ School of Public Health, Jilin University, Changchun, China; ^4^ Science for Life Laboratory, Department of Protein Science, School of Engineering Sciences in Chemistry, Biotechnology and Health (CBH), KTH Royal Institute of Technology, Solna, Sweden; ^5^ Department of Women’s and Children’s Health, Karolinska Institutet, Stockholm, Sweden

**Keywords:** estradiol (17ß-estradiol), estrogen receptor beta (ERß), estrogen receptor alpha (ERα), RNA-Seq - RNA sequencing, cistrome, proliferation

## Abstract

The two estrogen receptors ERα and ERβ are nuclear receptors that bind estrogen (E2) and function as ligand-inducible transcription factors. They are homologues and can form dimers with each other and bind to the same estrogen-response element motifs in the DNA. ERα drives breast cancer growth whereas ERβ has been reported to be anti-proliferative. However, they are rarely expressed in the same cells, and it is not fully investigated to which extent their functions are different because of inherent differences or because of different cellular context. To dissect their similarities and differences, we here generated a novel estrogen-dependent cell model where ERα homodimers can be directly compared to ERβ homodimers within the identical cellular context. By using CRISPR-cas9 to delete ERα in breast cancer MCF7 cells with Tet-Off-inducible ERβ expression, we generated MCF7 cells that express ERβ but not ERα. MCF7 (ERβ only) cells exhibited regulation of estrogen-responsive targets in a ligand-dependent manner. We demonstrated that either ER was required for MCF7 proliferation, but while E2 increased proliferation *via* ERα, it reduced proliferation through a G2/M arrest *via* ERβ. The two ERs also impacted migration differently. In absence of ligand, ERβ increased migration, but upon E2 treatment, ERβ reduced migration. E2 *via* ERα, on the other hand, had no significant impact on migration. RNA sequencing revealed that E2 regulated a transcriptome of around 800 genes *via* each receptor, but over half were specific for either ERα or ERβ (417 and 503 genes, respectively). Functional gene ontology enrichment analysis reinforced that E2 regulated cell proliferation in opposite directions depending on the ER, and that ERβ specifically impacted extracellular matrix organization. We corroborated that ERβ bound to cis-regulatory chromatin of its unique proposed migration-related direct targets ANXA9 and TFAP2C. In conclusion, we demonstrate that within the same cellular context, the two ERs regulate cell proliferation in the opposite manner, impact migration differently, and each receptor also regulates a distinct set of target genes in response to E2. The developed cell model provides a novel and valuable resource to further complement the mechanistic understanding of the two different ER isoforms.

## Introduction

Estrogen is important for the development of both female and male reproductive systems and for female secondary sex characteristics (1). Estrogen also impacts normal physiological functions, including metabolism, the immune system, and inflammatory responses (2), and some cancers. For example, estrogen drives growth of the hormone-sensitive form of breast cancer but reduces the incidence of colorectal cancer (3). Endogenous estrogens include estrone (E1), estradiol (E2), estriol (E3), and estetrol (E4), of which E2 is the most potent and prevalent in pre-menopausal females.

The biological functions of estrogens are mediated by estrogen receptors (ERs). They include the nuclear receptors ERα (encoded by the *ESR1* gene) and ERβ (encoded by *ESR2*). ERα and ERβ can form both homo- or heterodimers, bind DNA directly or tether to other transcription factors, and regulate target genes. They show a high degree of homology, especially in the DNA-binding domain (96%) but are relatively divergent in their terminal domains. The terminal domains interact with other proteins (including coregulators) which may impact transcriptional regulation considerably. The biological effects of ERα and ERβ have been reported to be both overlapping and distinct, and sometimes inverse (4). Knockout of either receptor in rodents generates infertile (ERα in mice and rats, ERβ in rats) or subfertile (ERβ in mouse) characteristics, along with subtle differentiating effects (e.g., on obesity, metabolism, tumor developments) (reviewed in (5)). However, to mechanistically compare the two receptors have been difficult. They are normally expressed in different cell types where ERα is highly expressed in female reproductive tissues (endometrium, cervix, uterine, vagina, and breast) and in some non-reproductive tissue (e.g., skeletal myocytes and liver hepatocytes) according to mRNA and protein levels (6, 7). ERβ, on the other hand, is expressed at lower levels and has been difficult to study, in part because of non-specific antibodies. It is expressed in granulosa cells of the ovary, cells of male reproductive tissues (early and late spermatids and spermatocytes of the human testis), adrenal gland, and some immune cells, according to mRNA and protein level (6, 7). They are rarely expressed in the same cell. Further, ERβ is not expressed in any known cell lines, and therefore, mechanistic and functional studies have been performed by introducing ERβ exogenously. Few studies have compared ERα with ERβ in the same cell type, and those that have, have either expressed both receptors exogenously in cells that are not innately estrogen responsive (e.g., HeLa cells), or added ERβ to estrogen-responsive ERα-expressing cells (e.g., MCF7 and T47D) (8, 9). The former rarely generate an ERβ protein that is estrogen-responsive in terms of transcriptional regulation of endogenous genes, and the latter is not able to fully separate the role of ERβ homodimer from ERαβ dimer.

A majority of breast tumors are estrogen dependent and overexpress ERα. ERα is the target of endocrine therapy and functions as a treatment-predictive biomarker. Consequently, the role of ERα in breast cancer has been thoroughly investigated and studies have shown that ERα can promote breast cancer cell proliferation. Mechanistically, its chromatin binding, cofactor interaction, and gene regulatory mechanism have been well characterized (reviewed in (10, 11)). Thus, well-characterized breast cancer models are available and highly suitable systems for functional and mechanistic comparisons between ERα and ERβ. ERβ, however, is not generally expressed in breast cancer (6). Its introduced expression in breast cancer cell lines has demonstrated that it has characteristics of a tumor suppressor and functions differently from ERα (12). The MCF7 cell line is the most well-characterized and established model to study ERα transcriptional activation and function (11). Gene expression studies in MCF7 have described that exogenous addition of ERβ alters the estrogen-mediated gene regulation (9, 13, 14), and studies of the ERs chromatin-wide binding pattern have shown that while they share a large fraction of binding sites (including at ERE motifs) they also have distinct binding patterns (15, 16). However, these models could not differentiate the activity of ERαβ heterodimers from that of ERβ homodimer and a pure comparison between ERα and ERβ-regulated genes in an estrogen-sensitive context has not been achieved.

Because ERβ has been found to have antiproliferative effects, some studies have utilized a tetracycline (Tet)-regulated transactivator (Tet-Off) system for its exogenous expression. By transfecting a vector with *ESR2* under the control of a Tet-responsive promoter, the ERβ gene can be inserted and its expression induced only when needed (by removing Tet from the media). This model has been used previously to study the cistrome of ERαβ heterodimers and corresponding transcriptome (9, 17).

Based on such previously generated MCF7 Tet-Off ERβ cell line model (17), we here describe the deletion of ERα expression using CRISPR-Cas9, and the generation of a new MCF7 cell model that express ERβ but not ERα. This enables the direct comparison of ERα and ERβ homodimers in the same cellular (MCF7) background. In this study, we characterize their different responses to E2 in respect of proliferation, migration, and transcriptome-wide gene expression. We provide novel and valuable mechanistic and functional information, identify specific similarities and differences of ERα and ERβ, along with an experimental resource to complement the understanding of their roles and their specific molecular mechanism.

## Materials and Methods

### Cell Lines and Treatments

The stable MCF7 Tet-Off ERβ cell line were previously generated and is available in our lab (17). The cells express ERβ in the absence of Tet. These modified MCF7 cells were cultured in Dulbecco’s Modified Eagle’s Medium (DMEM) supplemented with 10% fetal bovine serum (FBS) and 1% Penicillin/Streptomycin (P/S) at 37°C and 5% CO_2_.

### CRISPR-Cas9-Mediated ERα Knockout

**Figure 1:**
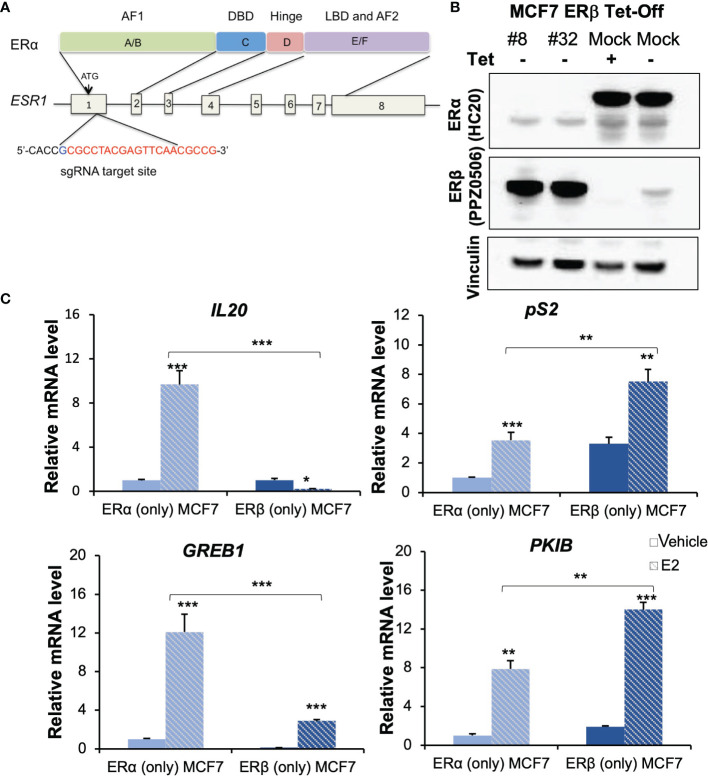
Generation and characterization of ERβ (only) MCF-7 cells. **(A)** Domain structure of ERα (upper) and genomic structure of *ESR1* gene (bottom). The sgRNA target site is located at 5’-end of exon 1 and the 20-nt guide sequence is indicated in red letters. **(B)** ER expression was validated for the indicated receptors by Western blot analysis, using vinculin as loading control. Lane 1-2: ERβ (only) #8 and #32 clones, lane 3: ERα (only) cells (mock, in presence of Tet) and lane 4: showing induction of ERβ (mock, 20h after removal of Tet). **(C)** mRNA levels of *IL20*, *pS2, GREB1* and *PKIB* were measured by qPCR in ERα (only) and ERβ (only, #32) following treatment with vehicle or E2. Data is represented as means + SD (n=3) and analyzed using two-way ANOVA followed by Bonferroni test, *P < 0.05, **P < 0.01, ***P < 0.001.

A single guide RNA (sgRNA) for ERα exon1 (CACCGCGCCTACGAGTTCAACGCCG, **Figure 1A**) was designed using the CRISPR gRNA design tool (https://www.atum.bio/eCommerce/cas9/input) and cloned into pSpCas9n (BB)-2A-GFP (PX461) vector (Addgene plasmid 48140) following a standard protocol (18). Transfection into the MCF7 Tet-Off ERβ cells was carried out using Lipofectamine 2000 (Invitrogen). Cells were suspended and cultured in DMEM medium with 10% FBS and 1% P/S and incubated at 37°C with 5% CO_2_. After 24 h, cells were sorted by fluorescence-activated cell sorting (FACS) to capture cells with high green fluorescent protein (GFP) signals, and these were seeded as single cells into 96-well plates and cultured. PX461 empty-vector transfected and sorted single-cell clones were used as negative controls (mock). The resultant single-cell colonies were sequenced and colonies with successful knockouts were validated by Western blot **Figure 1B**, **Supplementary Figure 1A**).

### Western Blot

Tet was added or removed 48 h before harvesting cells, to stop or induce ERβ expression. MCF7 Tet-Off ERβ cells with Tet treatment generated ERα-only cells, here denoted MCF7 (ERα only). MCF7 Tet-Off ERβ CRISPR-ERα with Tet treatment generated cells without either ER, here denoted MCF7 (no ER). MCF7 Tet-Off ERβ-CRISPR-ERα in absence of Tet generated cells with expression of ERβ and not ERα, here denoted MCF7 (ERβ only). Western blotting was performed as described elsewhere (19) with primary antibodies anti-ERα (HC-20; rabbit polyclonal sc-543; Santa Cruz Biotechnology, RRID: AB_631471, dilution 1:600; and 1D5; mouse monoclonal, Thermo Fisher, RRID: AB_10986080, dilution 1:500), anti-ERβ (PP-PPZ0506-00, mouse monoclonal, Perseus Proteomics, RRID: AB_604962 dilution 1:1000), anti-vinculin as loading control (H-10; mouse monoclonal sc-25336, Santa Cruz Biotechnology, RRID: AB_628438, dilution 1:200), and secondary anti-mouse antibody (NA931, dilution 1:5000) from Sigma-Aldrich or secondary anti-rabbit antibody (7074S, dilution 1:5000) from Cell Signaling Technology.

### Quantitative PCR

qPCR was performed using Fast SYBR Green Master Mix (Applied Biosystems) as previously described (20).

### Cell Proliferation Assay

Cell proliferation was measured at indicated time points and treatments using the WST-1 reagent (Roche Applied Science) following protocols from the manufacturer.

### Clonogenic Cell Survival Assay

MCF7 (parental cells), MCF7 (ERα only), MCF7 (ERβ only), and MCF7 (no ER) were seeded in 12-well plate with 2000 cells per well. Following culture for 8 days in normal DMEM medium, cells were fixed (acetic acid/methanol 1:7), stained (0.5% crystal violet for 2 h), and quantified by measuring by fluorescence of extracted crystal violet (10% cold acetic acid) at OD590nm.

### Flow Cytometry

Flow cytometry was carried out to analyze the cell cycle. MCF7 (ERα only) or MCF7 (ERβ only) were grown in 2.5% DCC-FBS medium in absence of ligands for 72 h, followed by E2 (10nM) or vehicle treatment for 24 h. Cells were harvested by trypsinization and fixed with 70% cold ethanol for 30 min. After washing the fixed cells with cold PBS, the cells were stained with 50 μg/ml propidium iodide supplemented with RNase A (Sigma) for 30 min at 37°C, followed by flow cytometry analysis using a FACS Calibur flow cytometer (BD Biosciences). Cell cycle analysis was performed using CELLQuest program (BD Biosciences).

### Migration Assay

The migration assay was performed with the Culture-Insert 4 Well µ-Dish (80466, Ibidi). The µ-Dishes were placed in 6-well plate, cells were seeded in different chambers, and incubated for 24 h. The cells were then cultured under either full serum conditions, or low-serum non-estrogenic conditions (DMEM with 2.5% DCC-FBS, without phenol red for 72 h) and the µ-Dishes were gently removed with sterile tweezer. Cells cultured under non-estrogenic conditions were treated with E2 (10 nM) or vehicle. Cell migration was determined after 24 h and 48 h by measuring the gap and comparing to the initial area using Image J.

### RNA-Sequencing and Analysis

MCF7 (ERα only) or MCF7 (ERβ only) cells were grown in absence of ligands (2.5% DCC-FBS medium) for 72 h, followed by E2 (10 nM) or vehicle treatment for 24 h. Total RNA from three biological replicates of each condition were extracted using RNeasy Plus Mini Kit (QIAGEN). Library constructions were performed and sequenced on an Illumina HiSeq 2000 following the manufacturer’s protocol at the Bioinformatics and Expression Analysis core facility (BEA, Karolinska Institutet, Sweden). The generated sequences were aligned to the human genome reference (GRCh38) using TopHat (v2.0.12). Read counts were obtained using HT-seq (v0.6.1) and differential expression analysis was performed using the DESeq2 workflow. Cut offs for statistical significance (FDR ≤ 0.05), fold change (absolute value of logFC ≥1), and expression (RPKM ≥ 1 in either treatment group) were applied in order to identify differentially expressed genes. Analysis of enrichment of Gene Ontology biological processes among differentially expressed genes was carried out with the online tool Database for Annotation, Visualization, and Integrated Discovery (DAVID, https://david.ncifcrf.gov/), and Ingenuity Pathway Analysis (IPA) was used for analyzing molecular and cellular functions, with P-value ≤0.05 considered significant. Data is deposited in NCBI’s Gene Expression Omnibus (GEO) (GSE182431).

### ChIP-Seq Comparison

Cistrome data for ERα was downloaded from GEO (GSE128208) (21) and data for ERβ was downloaded from GSE149979 (22). The promoter region was defined by -1kb to 100bp distance from transcription start sites (TSS). *De novo* motif analysis was performed by within 200bp of binding peaks using HOMER.

### ChIP-qPCR

ChIP was performed as previously described (22). In brief, MCF7 (ERβ only) and MCF7 cells co-expressing ERα and ERβ (MCF7 Tet-Off ERβ cells in absence of Tet) were cultured in 15-cm plates until 80% confluent. Before ChIP, the cells were starved with DMEM medium (without phenol red or FBS) for 24 h and then treated with E2 (10 nM) for 2 h. Cells were cross-linked with 1% formaldehyde and quenched by glycine (0.125M). After harvest, the cells were washed by lysis buffer and sheared by sonication. The sheared chromatin was immunoprecipitated with ERβ antibody (R&D system, PP-PPZ0506-00; mouse monoclonal, RRID: AB_604962) and Protein G Dynabeads (cat no: 10004D, Invitrogen). The DNA was purified with QIAquick PCR purification kit (Qiagen, cat no: 28104). qPCR was performed with Fast SYBR Green Master Mix (Applied Biosystems).

### Statistical Analysis

The data that is presented are representative of two or three independent experiments. Each experiment include duplicate or triplicate technical replicates. For comparing differences between two groups when data was normally distributed, unpaired two-tailed Student’s t-test was used to test statistical significance. When data was not normally distributed (n<5), nonparametric test was used. Between three or more groups, one-way ANOVA was used, and for data with two variables (treatment and genotype), two-way ANOVA followed by Bonferroni test were used. P ≤ 0.05 was considered statistically significant.

## Results

### Generation of ERβ+/ERα- MCF7 Cells

The ERs exhibit cell context specific behaviour. To identify similarities and differences between the functions of ERα and ERβ, respectively, it is necessary to compare their activity in cells of the exact same background. Further, it is important that the cells are estrogen-sensitive, and that the ERs generate transcriptional regulation of endogenous genes in response to estrogen. The regulatory activity of ERα in the Luminal A (ERα+, PR+, HER2-) subtype of breast cancer is one of the most studied gene regulatory mechanisms, and this type of breast cancer cells are therefore an excellent estrogen-responsive cell model to use for comparison of the two homologues. The Luminal A subtype MCF7 cell line is the most widely used model for studies of ERα and is the cell line from which this receptor was originally cloned. Therefore, we selected this model to characterize exactly how similar and different ERβ is from ERα. MCF7 Tet-Off-inducible ERβ expressing cells (endogenous ERα and inducible exogenous ERβ) were previously generated and available in our lab. Based on this, we generated a novel MCF7 cell model that express ERβ but not ERα. We performed ERα knockout by CRISPR-Cas9 editing **Figure 1A**). The sgRNA, directed towards a site in exon1 of the *ESR1* gene, was GFP tagged, cloned, and transfected into MCF7 Tet-Off ERβ cells. GFP-expressing single cells were grown into colonies and evaluated by Western blot. Among more than 100 such single-cell clones of CRISPR-Cas9 edited cells, we found two, #32 and #8, that had no ERα expression and where ERβ was still induced in absence of Tet **Figure 1B**, **Supplementary Figure 1A**). We chose clone #32 to do further exploration and clone #8 to confirm the function of ERβ. Meanwhile, clones transfected with the CRISPR-Cas9 control vector (without sgRNA) maintained expression of endogenous ERα both in presence and absence of Tet **Figure 1B**). To corroborate that *ESR1* has been edited by sgRNA, we extracted the genomic DNA and conducted TA cloning and DNA sequencing. As shown in **Supplementary Figure 1**, the editing succeeded in introducing 1-base or 2-base frameshifting insertions in the *ESR1* allele, which resulted in absence of ERα translation and corresponding protein. Hence, in presence of Tet these cells do not express any ER, and in the absence of Tet they express ERβ only. As control, we used the MCF7 Tet-Off inducible ERβ cells, which in presence of Tet express only endogenous ERα. We also compared this control to parental MCF7 cells, to ensure they maintained their innate ERα activity.

Next, to evaluate and confirm functionality and estrogen response of the induced ERβ homodimers in MCF (ERβ only), we selected four well-known ERα-E2 upregulated genes (*IL20, pS2*, *GREB1, PKIB)* that also had reported ERβ chromatin-binding sites and considered to be targets of both receptors (23). As expected, these genes were upregulated by E2 in MCF7 (ERα only) cells. In cells lacking both ERs (ERα knockout cells treated with Tet), none of these genes were regulated by E2 (**Supplementary Figure 1C**). In cells expressing only ERβ (ERα-/ERβ+), three of the genes (*pS2, GREB1*, and *PKIB)* were upregulated by E2, whereas one, *IL20*, was downregulated **Figure 1C**, clone #32). We noted a similar pattern in the #8 clone, with upregulation of *pS2, GREB1*, and *PKIB*, and lack of upregulation of IL20 by E2 (**Supplementary Figure 1C**). We also noted effects on their basal level expression (in absence of E2), depending on which ER was expressed. Presence of ERβ significantly increased the level of *pS2* and *PKIB*, and decreased the levels of *IL20* and *GREB1* in both clones. Notably, IL20 was nearly absent in clone #8, and no further ERβ-mediated downregulation could be noted upon treatment with E2. These results confirmed the deletion of ERα activity (cells lacking both ERs did not regulate these four genes in response to E2) and that the introduced ERβ was functional and showed both ligand-independent and ligand-dependent effects.

### Functional Impact of ERα and ERβ Homodimers on Cell Proliferation

**Figure 2:**
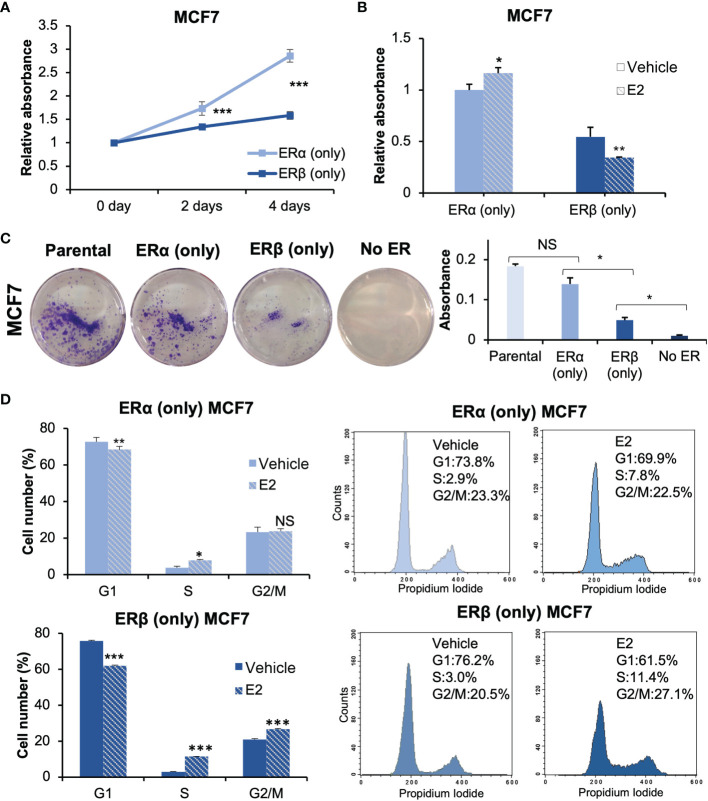
ERα and ERβ impact cell proliferation differently. **(A)** Cell proliferation of ERα (only) or ERβ (only) MCF7 cells was measured using WST-1 assay. Cells were grown in full-serum medium and measured at day 0, 2, and 4. Absorbance at day 0 was used for normalization. **(B)** The cell lines were pre-cultured under non-estrogenic and serum-starved conditions, followed by E2 or vehicle treatment and measured by WST-1 assay at day 4. Absorbance of ERα (only) MCF7 cells with vehicle stimulation was used for normalization. **(C)** For clonogenic assay, the cells were cultured in full-serum medium for 8 days. Extracted crystal violet was used for quantification (right). **(D)** Flow cytometry analysis of cell cycle progression of ERα (only) or ERβ (only) MCF7 cells (right) and corresponding quantitation of cell cycle distribution (G1, S and G2/M, left). Cells were grown in 2.5% DCC-FBS medium for 72 h, followed by treatment of E2 or vehicle for 24 h. Data is illustrated as means ± SD (n=3). A, B, D were analyzed using two-way ANOVA followed by Bonferroni test; C was analyzed using one-way ANOVA. *P < 0.05, **P < 0.01, ***P < 0.001, NS, not significant.

ERα is known to be essential for estrogen-dependent breast cancer cell proliferation (24), whereas ERβ appears to have an antiproliferative function although conflicting data exist regarding its role (14). To investigate the roles of respective homodimer, we performed cell proliferation assays **Figure 2**). First, MCF7 cells with either ERα or ERβ expression were cultured in full-serum medium, and proliferation was measured at day 0, 2, and 4 **Figure 2A**). MCF7 cells with either ER proliferated, but ERα (only) grew significantly faster than ERβ (only) cells. MCF7 cells without either ER did not proliferate. Next, we investigated the proliferative response to E2 treatment **Figure 2B**). Cells were cultured under non-estrogenic and low serum conditions. Following E2 or vehicle treatment, proliferation was measured after 4 days. In absence of E2, cells with ERα (only) grew better than cells with ERβ (only). In response to E2, ERα (only) cells increased their proliferation, whereas ERβ (only) cells significantly reduced their proliferation in a ligand-dependent manner. To control for possible impacts by the Tet-Off system or CRISPR-Cas9 editing, we compared cell proliferation between parental MCF7 cells (ERα only, no Tet), MCF7 Tet-Off ERβ mock (CRISPR empty-vector transfected control) in presence of Tet, and MCF7 Tet-Off ERβ in presence of Tet (all expressing ERα only, **Supplementary Figure 2A**). All three types of cells proliferated with similar speed, indicating that neither the Tet-Off system nor CRISPR-Cas9 transfection significantly impacted the cell proliferation. We also performed a clonogenic cell survival assay **Figure 2C**, **Supplementary Figure 2B**). ERα enabled a higher degree of colony formation compared to ERβ, and ERβ allowed for more colony formation than no ER which did not form colonies. Thus, either ER was necessary for colony forming ability and for proliferation. Finally, we performed analysis with flow cytometry to investigate their precise impact on the cell cycle **Figure 2D**). Upon stimulation with E2, ERα increased the fraction of cells in S phase (7.8% vs 2.9%) and did not significantly impact the proportion of cells in the G2/M phase. ERβ homodimers induced an even stronger accumulation of cells in S phase upon E2 stimulation (11.5% vs 3.0%), but in addition caused cells to accumulate in G2/M phase (26.6% vs 20.9%) resulting in a lower proportion of cells in G1 (76.2% to 61.5%). This indicated that both receptors induced S phase in response to E2, but that ERβ also mediated G2/M arrest and hence inhibited cell proliferation. In conclusion, the results clearly demonstrates that ERα and ERβ homodimers impact cell proliferation differently. In these estrogen-dependent MCF7 cells, while either ER (including ERβ) is essential for cell proliferation (i.e., similar functions), ERα sustains proliferation to a significantly higher degree and E2-ERβ reduces cell proliferation through G2/M arrest.

### The Roles of ERα and ERβ Homodimers in Cell Migration

**Figure 3:**
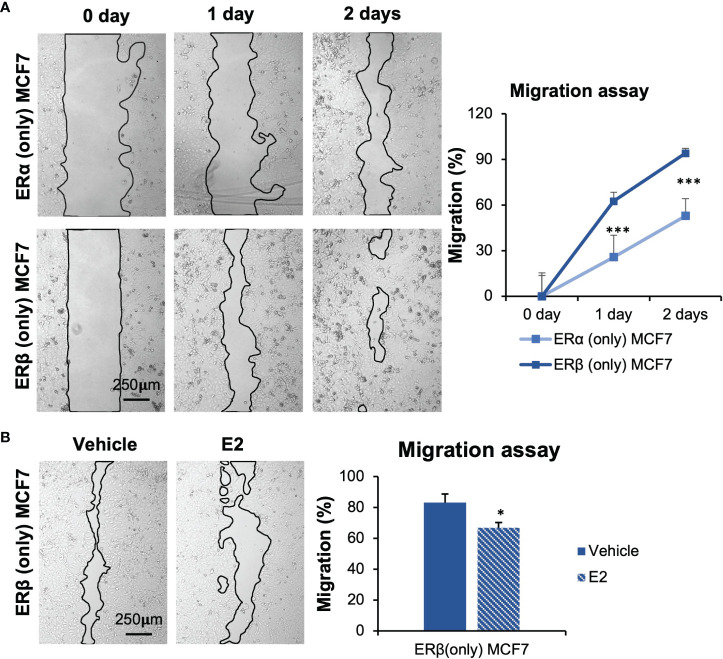
ERα and ERβ impact cell migration differently. **(A)** Wound healing assay was performed using inserts (µ-Dish). Cells were cultured in full-serum medium, and pictures were taken at day 0, 1, and 2 after inserts were removed. Area after migration was measured with ImageJ. The initial area at day 0 for each cell line was used for normalization. **(B)** Impact of E2 treatment in ERβ-expressing MCF7 cells was measured. Cells were seeded in presence of inserts, starved for 72 h, inserts were removed, and cells were treated with E2 or vehicle. Pictures were taken after 2 days. Data is presented as means ± SD (n=6-8). A was analyzed by two-way ANOVA followed by Bonferroni test, B was analyzed by Student’s t-test. *P < 0.05, ***P < 0.001.

During the cell model establishment, we found that MCF7 cells with ERβ (only) expression exhibited a different morphology compared to cells with ERα (**Supplementary Figure 2C**). ERβ (only) MCF7 cells appeared larger in size and irregularly shaped compared to ERα (only) cells. Different morphologies may indicate that cell migration is impacted. To explore effects on cell migration, we performed migration assay **Figure 3**). The results showed that MCF7 ERβ (only) cells, under full serum conditions, migrated significantly faster than those with ERα only **Figure 3A**, **Supplementary Figure 2D**). This is despite the slower proliferation of ERβ (only) cells. Also under non-estrogenic culture conditions, cells with ERβ migrated faster than cells with ERα, but following E2 treatment (10 nM, 48 h), ERβ (only) cells reduced their migration **Figure 3B**, **Supplementary Figure 2E** lower panel). Cells with ERα (only), did not significantly change migration in response to E2 (**Supplementary Figure 2E**, upper panel). To be noted, ERα (only) MCF7 cells were treated with E2 for only 24 h, as the E2-promoted cell proliferation caused the cells to become too confluent after this. We conclude that, within the same cellular context, ERβ (in absence of added ligand) increases migratory capacity compared to ERα, but in response to E2 (10 nM), ERβ but not ERα reduces migration.

### Identification of ERα- and ERβ-Specific Transcriptomes

**Figure 4:**
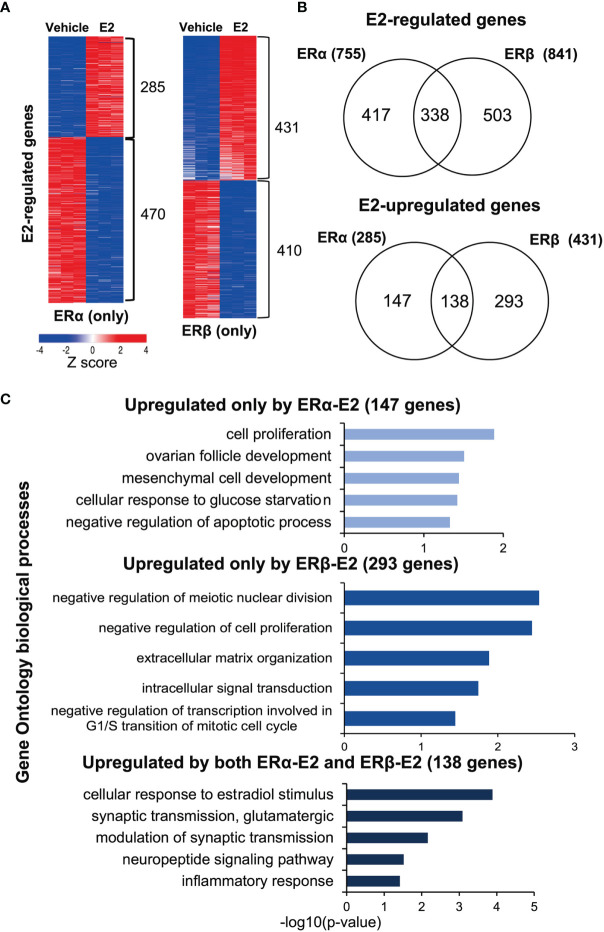
ERα and ERβ regulate the transcriptome differently. **(A)** Heatmap illustrating E2-regulated gene expression profiles by ERα (left) and ERβ (right), as determined by RNA-seq. Red indicates higher expression, blue lower. The heatmap was generated using the web-based tool Morpheus (https://software.broadinstitute.org/morpheus/) and the gene expression data (log2fold change) were normalized by Z-score. **(B)** Overlap of all E2-regulated genes (top), and E2-upregulated genes specifically (bottom) by ERα or ERβ. **(C)** Enrichment analysis of biological functions related to the E2-upregulated genes (corresponding to the groups in B, lower panel) for ERα (147 genes, top), ERβ (293 genes, middle), and by both receptors (138 genes, lower graph) using DAVID.

To identify the transcriptome-wide estrogen response through ERα or ERβ homodimers, respectively, we performed RNA-seq. Hormone-deprived MCF7 ERα (only) or MCF7 ERβ (only) cells were treated with either E2 or vehicle (24 h). The E2-mediated transcriptional profiles were generated in triplicates and clustered into significantly up- or downregulated genes according to the ER isoform **Figure 4A**). In ERα (only) MCF7 cells, 755 genes were regulated by E2 stimulation, of which more genes were downregulated (470) than upregulated (285 genes). The E2-regulated genes in the MCF7 (ERα only) cells overlapped (73%) with previously reported regulations of parental MCF7 cells (GSE148276, applying FDR ≤ 0.05 for both analysis) (25), confirming that the ERα (only) MCF7 cells (Tet-Off ERβ with Tet treatment) retained the parental functions of ERα. A similar number of genes (841) were detected as regulated by ERβ upon E2 treatment, with about equal numbers being up- and downregulated (431 and 410, **Figures 4**, right). However, only about a fifth of all genes were detected as regulated by both ERs (338 genes out of 1596 E2-regulated genes, or 21%). Of these, however, nearly all (329/338) were regulated in the same direction (138 upregulated, 191 downregulated) by both ERα and ERβ in response to E2 (**Figure 4**, **Supplementary Figure 3A**). Only 9 genes showed opposite responses under E2 stimulation in the presence of ERα or ERβ. ERα induced and ERβ repressed 4 genes (*IL20, PEG10, RASGRP1, RAB30*) and ERβ induced and ERα repressed 5 genes (*FOXI1, RAB19, GLRX, P2RY2, ANXA9*). The RNA-seq data thereby was in accordance with the qPCR generated data of opposite regulation of *IL20* (clone #38, **Figure 1**).

Thus, despite the two ERs having highly conserved DNA binding domains and being investigated within the exact same cellular context (MCF7 cells), most genes were regulated exclusively by either ERα (147 up, 279 down) or ERβ (293 up, 219 down; **Figure 4**, **Supplementary Figure 3A**). To understand the functional impact of these isoform-specific E2-mediated effects, we performed functional gene annotation and enrichment analysis using DAVID (**Figure 4**, **Table 1**, **Supplementary Figure 3B**). We first investigated the genes that were similarly upregulated by ERα and ERβ in response to E2 (138 genes, **Figure 4**, bottom). Here, expected functions were overrepresented, including cellular response to estradiol stimulus (incl. *GPER1, NRIP1*) and inflammatory response (incl. *IL1RAP*), supporting important and well-known commonalities between the two receptors’ functions. Next, the functional annotations of genes upregulated exclusively by ERα (147 genes) were investigated **Figure 4C**, top). The most enriched function was cell proliferation (incl. *MYC, BCL2*), which is in accordance with its well-characterized function. Notably, ERα-regulated genes were also enriched for negative regulation of apoptotic process [incl. *XBP1* which ERα has recently been shown to mediate alternative splicing of (26)]. Finally, the genes upregulated exclusively by ERβ (293 genes, **Figure 4**, middle) were investigated. These were specifically enriched for negative regulation of meiotic nuclear division, negative regulation of cell proliferation, and negative regulation of transcription involved in G1/S transition of mitotic cell cycle. This is highly in agreement with the findings of its proliferative functions described above (**Figure 2**). Important gene regulations in these categories include the cell growth regulator that controls cell cycle G1 progression *CDKN2D* and the regulator of cell cycle progression *E2F7*. The upregulation of CDKN2D can induce G2/M arrest (27) and the increased E2F7 can drive cells from G1 to S phase, which both are consistent with the results (**Figure 2**) that ERβ reduced proportion in G1 phase and increased proportion in S and G2/M phase following E2 treatment (28). Also, functions within intracellular signaling (e.g., *JAK2, MAP4K3*) and extracellular matrix formation (e.g., laminins *LAMA3, LAMC2*, and collagen *COL18A1*) were enriched for among ERβ-upregulated genes.

**Table 1:** GO biological processes analysis for E2-upregulated genes.

By ERα	Genes	P value	Gene names
Cell proliferation	5	0.01	BCL2, MYC, POLR3G, FOXC1, MCM10
Ovarian follicle development	3	0.03	BCL2, MYC, FOXC1
Mesenchymal cell development	2	0.04	BCL2, FOXC1
Cellular response to glucose starvation	3	0.04	BCL2, XBP1, SLC2A1
Negative regulation of apoptotic process	8	<0.05	BCL11B, BCL2, FCMR, GRK5, HCK, MYC, XBP1, ADORA1
**By ERβ**			
Negative regulation of meiotic nuclear division	3	0.003	FBXO5, LIF, RPS6KA2
Negative regulation of cell proliferation	14	0.004	E2F7, JAK2, KISS1, LIF, WNT9A, AZGP1, CHD5, COL18A1, CDKN2D, EREG, RPS6KA2, SULT2B1, TPBG, ZNF503
Extracellular matrix organization	9	0.01	ADAMTSL5, CD44, KAZALD1, CCDC80, COL18A1, FGG, FN1, LAMA3, LAMC2
Intracellular signal transduction	12	0.02	JAK2, TNIK, DGKZ, DNMBP, HSPB1, MAP4K3, NRG3, PPP1R1C, RPS6KA2, SCG2, SGK1, TNS1
Negative regulation of transcription involved in G1/S transition of mitotic cell cycle	2	0.04	E2F1, E2F7
**By both ERs**			
Cellular response to estradiol stimulus	5	0.0001	GPER1, ITGA2, NRIP1, SSTR2, ZNF703
Synaptic transmission, glutamatergic	4	0.0008	CNIH2, GRIK3, GRIK4, SLC1A4
Modulation of synaptic transmission	4	0.007	GPER1, GRIK3, GRIK4, SLC7A11
Neuropeptide signaling pathway	4	0.03	GAL, NXPH3, NPY1R, SSTR2
Inflammatory response	7	0.04	GPER1, GPR68, C5AR2, GAL, IL1RAP, LOXL3, SERPINA3

Among the genes repressed by both ERα and ERβ (191 genes, **Supplementary Figure 3B**), functions relating to cell migration, cell adhesion, and wound healing were enriched for. Also this was consistent with the E2-mediated phenotype of suppressed cell migration through ERβ, and perhaps also to the non-significant trend noted in ERα (only) cells. Genes specifically downregulated by ERα included genes linked to negative regulation of cell proliferation, the apoptotic process, and migration, as well as epithelial cell differentiation. Genes specifically downregulated by ERβ were involved in response to wounding (related to cell migration) and epithelial cell differentiation, and also the Wnt signaling pathway. We further used the Ingenuity Pathway Analysis to investigate molecular and cellular signaling pathways (**Supplementary Figure 4**). This generated similar results, but also identified that ERα-E2 upregulated genes were related to cell morphology, and ERβ-E2 regulated genes to protein synthesis. Overall, this data offers clear molecular underpinnings to their different functions within cell proliferation (**Figure 2**), migration (**Figure 3**), and morphology (**Supplementary Figure 2C**).

### Cistromes Support the Distinct Roles of ERα and ERβ

**Figure 5:**
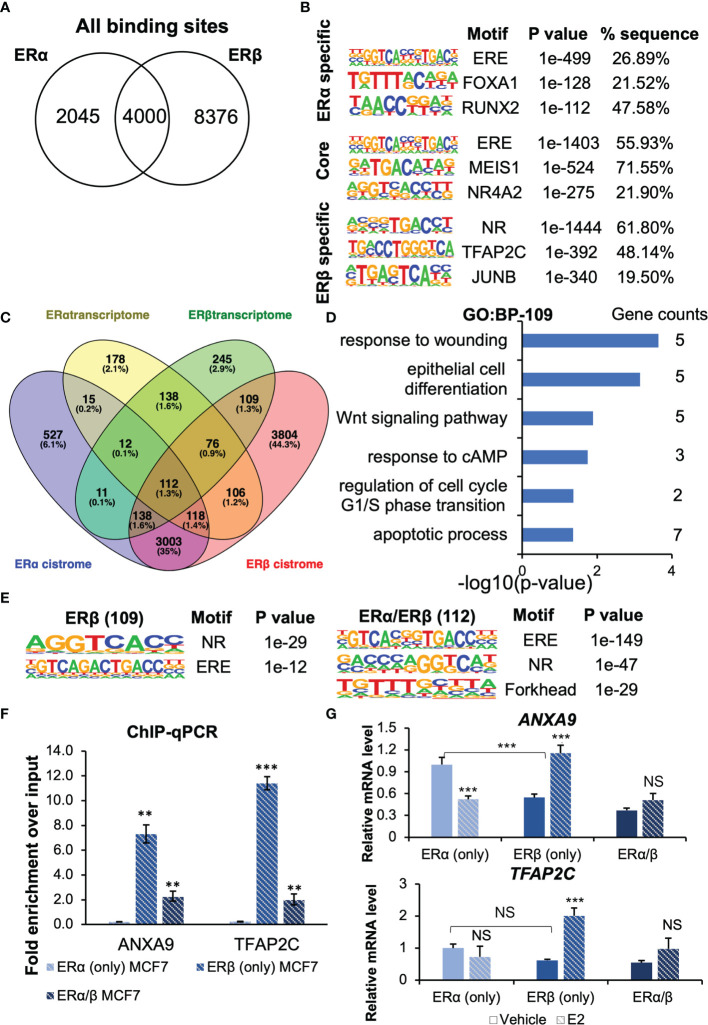
The ERs cistrome and transcriptome. **(A)** Venn diagram comparing ERα and ERβ binding sites. **(B)** Top-3 enriched motifs in ERα-specific, common core, and ERβ-specific cistrome. **(C)** Venn diagrams of ERα and ERβ transcriptome and cistrome data from MCF7 cells identify 109 genes regulated uniquely by ERβ through both chromatin-binding and transcriptional regulation, but only 30 genes that are unique for ERα. **(D)** Enrichment analysis of biological functions for the 109 uniquely ERβ-regulated genes. **(E)** Top-3 enriched motifs among ERβ-specifically regulated genes, and those regulated by both ERs. **(F, G)** Confirmation of ER binding and regulation of *ANXA9* and *TFAP2C* with ChIP-qPCR and qPCR. Data is shown as means ± SD (n=3), and analyzed by two-way ANOVA followed by Bonferroni test. *P* < 0.05, **P < 0.01, ***P < 0.001, NS, Not significant.

Our group has previously identified the cistrome of ERβ (in presence of ERα) in the MCF7 Tet-Off ERβ cells (22). We here compared this ERβ dataset with an MCF7 parental ERα cistrome dataset (21). Among the over 14 000 chromatin sites bound by either receptor, 4000 could be bound by both ERα and ERβ **Figure 5A**). Among this ER “core” cistrome, the ERE motif was the most enriched binding sequence, as expected **Figure 5B**, middle). The binding sites that were specific for either ERα or ERβ were also enriched for ERE (or NR) motifs. In addition, ERα bound more often to locations with FOXA1 and RUNX2 motifs, whereas ERβ was more enriched at TFAP2C and JUNB motifs. By overlapping the cistromes of ERα and ERβ (annotated by the gene located nearest to each chromatin-binding sites), with respective homodimer transcriptome (our study), we found that about a third (257 out of 755, or 34%) of ERα-E2-regulated genes had an ERα-binding chromatin site located nearest to it, and that as much as half (435 out of 841, or 52%) of ERβ-E2-regulated genes held corresponding ERβ-binding sites **Figure 5C**). One fourth (109 genes) of these plausible ERβ direct target genes (435 genes) were not regulated by and not bound by ERα (**Figure 5C**). These unique ERβ-targets (109 genes) were enriched for functions within response to wounding, epithelial cell differentiation, and Wnt signaling pathway **Figure 5D**). To investigate whether different tethering factors could be impacting the different gene regulations, we repeated the motif analysis for the bound DNA by the genes which were *de facto* regulated (112 by both ERs, 109 by ERβ only, **Figure 5E**). However, we found no significant differences, only the ERE and NR motifs were significantly enriched among the unique ERβ-regulated genes.

To be noted, among the top-50 genes that were uniquely upregulated by ERβ (sorted by significance), as many as 34 (68%) had an ERβ binding site. Similarly, among the 5 genes that were upregulated by ERβ but downregulated by ERα, all but one had ERβ bound in cis-regulatory chromatin (incl. promoter/TSS area by ANXA9). ERα on the other hand, bound only one of these. It is known that the ERs can regulate genes through long-distance binding, but it is difficult to predict which gene is regulated. It does not need to be the nearest gene on the chromosome, because of chromatin looping. To predict regulated genes with high certainty, we restricted the analysis to chromatin bindings by the promoter regions (-1kb to 100bp from TSS). This resulted in 76 putative direct targets of ERβ that also were regulated at the transcript level. A large proportion of these (33 genes) were not regulated by ERα (**Supplementary Figure 5A**). The same analysis for ERα yielded 26 direct targets, of which 9 were not regulated by ERβ.

Finally, we investigated two targets regulated by ERβ in more detail. *ANXA9* which is related to metastasis (29–31), and the transcription factor activating enhancer-binding protein 2C (*TFAP2C*) that has been reported to decrease migration and invasion in pancreatic ductal adenocarcinoma and non-small cell lung cancer cells (32, 33). *TFAP2C* can also regulate ERα expression (binds to the *ESR1* promoter region) (34). *ANXA9* was upregulated by ERβ in our analysis and downregulated by ERα. It harbors an ERβ binding site its promoter region, but ERα does not bind cis-regulatory chromatin by this gene. We performed ChIP-qPCR with ERβ antibody in MCF7 (ERβ only) as well as in MCF7 cells that co-express both ERs (**Figure 5F**). We confirmed the binding of ERβ to the chromatin regions by *ANXA9* (AP2 motif) and *TFAP2C* (ERE motif), which was significantly enriched (compared to MCF7 ERα (only) negative control). This data clearly demonstrates that ERβ binds these sites as homodimer. Next, we performed RT-qPCR to investigate their transcriptional regulations in further detail **Figure 5G**) This showed that ERβ in absence of ligand reduced the expression of *ANXA9* but following E2 treatment, ERβ upregulated its level. In ERα (only) cells, E2 downregulated *ANXA9.* Consequently, when both receptors where present (ERα/β cells), the level in absence of ligand was reduced, and in response to E2 the ERs neutralized each other, and the impact was reduced. Similarly, we corroborated that ERβ bound to the *TFAP2C* promoter as a homodimer **Figure 5F**, bottom). Also here, presence of unliganded ERβ reduced its level, but E2 treatment increased its expression in a ligand-dependent manner **Figure 5G**). E2 *via* ERα did not significantly impact *TFAP2C* expression, although RNA-seq data had indicated upregulation. The cistrome data indicated a chromatin-binding site only for ERβ, which is in accordance with the qPCR data. Consequently, in cells expressing both receptors, the E2-mediated upregulation was less apparent. Thus, the E2-mediated upregulation of *TFAP2C via* ERβ is consistent with the E2-reduced migratory activity in ERβ (only) cells.

## Discussion

The aim of this study was to identify and characterize the similarities and differences in gene regulation and corresponding functionality of ERα and ERβ homodimers. It is to be noted that no breast cancer cell line expresses native ERβ, and while significant interest has been directed to the possibility of using ERβ as a target in breast cancer, current evidence does not support its expression in breast cancer cells of any subtype (6). Our aim with this study was not to assess a role for ERβ in the breast, but to achieve a mechanistic and functional understanding of the differences between the receptors.

Previous studies of ERβ have been performed either by expressing ERβ in ERα-expressing estrogen-responsive cell lines (e.g., MCF7) or by expressing ERβ in non-ER expressing, non-estrogen-responsive cells (e.g., colon cancer cell lines). However, the former alternative results in formation of ERα/β heterodimers, which functions in part similar to the ERα dimer (35), and this does not sufficiently enable a direct comparison between ERα and ERβ. The latter alternative generates homodimers that show ligand-dependent response in ERE-transactivation reporter assays, but usually does not result in estrogen-activated gene regulation of endogenous genes, as noted in multiple previous studies (23, 36, 37). Thus, for the purpose of a direct comparison of the homologues, including their estrogen-activated transcriptome, we generated a novel cell model constituting of MCF7 cells that express only ERβ. The CRISPR-Cas9 introduced frameshifting insertions on both alleles stopped the translation of ERα protein in MCF7 Tet-Off ERβ-inducible cells. Importantly, the resulting ERβ homodimer in the MCF7 background exhibited ligand-dependent E2-mediated transcriptional regulation of endogenous genes. Thereby, a direct comparison between their ligand-dependent activities could be compared at the transcriptome level along with corresponding functional impact.

While incompletely understood, previous studies have consistently shown that both receptors impact proliferation. The effect of ERβ has in general been reported to be antiproliferative, and in part been attributed its ability to counteract ERα through the formation of heterodimers (12, 38). Some studies, however, have reported that ERβ in parental MCF7 cells can increase cell growth (8, 14). We here confirmed that ERα and ERβ, as individual homodimers, had opposite roles on regulating proliferation of MCF7 cells in response to E2 stimulation. Clearly, ERα increases proliferation in response to E2 and ERβ reduces proliferation in response to E2 treatment by inducing a G2 arrest, consistent with previous studies (8, 38). However, we also found that while MCF7 cells without either ER could not maintain proliferation, the introduction of ERβ could enable proliferation. This demonstrates that also ERβ have a role in maintaining cell growth. The controversy on its role in proliferation may thus be related to ERβ having both proliferative and antiproliferative roles, and the outcome would be dependent on the conditions of the experiments (e.g., estrogenic conditions, controls, homodimers versus heterodimer). At the gene regulatory level, the differential impact of ERα and ERβ where enriched for genes with functions in cell proliferation. This included five genes (*MYC, FOXC1, BCL2, MCM10, POLR3G*) that were exclusively upregulated by ERα (**Table 1**). Among them, *MYC* is a well-known estrogen-responsive proliferative gene in breast cancer (39–41), which our current study finds is not regulated by E2 in ERβ (only) cells. Fourteen genes involved in ‘negative regulation of cell proliferation’ were exclusively upregulated by ERβ-E2 (e.g., *KISS1, E2F7, CDKN2D, WNT9A, JAK2, LIF*, **Table 1**). Among these, KISS1 inhibits both proliferation and metastasis (42, 43), and the Wnt ligand, WNT9A suppresses breast cancer cell proliferation and is a tumor suppressor of colorectal cancer (44, 45). WNT9A is a member of the WNT gene family and can decrease cellular proliferation (44, 46). The cyclin-dependent kinases inhibitor CDKN2D that can form a stable complex with CDK4 or CDK6 to block G1-S progression (47), E2F7 that can negatively influence cellular proliferation and impact response to DNA-damage (48–50), and JAK2 which is a negative regulator of ERα function (51), were also upregulated by ERβ and not regulated by ERα. LIF (leukemia inhibitory factor) is a member of the IL-6 cytokine family and can promote malignancy progression in some tumors, and have anti-neoplastic effects in others (52). Previous studies have reported the transcriptome of ERα and ERβ in Luminal A cell lines MCF7 or T47D when co-expressing ERβ with ERα (9, 13, 14, 16, 53–55). However, in these studies, the resulting transcriptome is mediated through a mix of ERαβ, ERαα and ERββ dimers. Still, specific gene regulations correlated well with previous analysis of ERβ co-expressed in ERα-positive breast cancer cells. For example, we have previously, in T47D-ERβ Tet-Off cells (co-expressing ERα and ERβ) found *MYC* to be upregulated by ERα and opposed upon introduction of ERβ (9), and *E2F7* to be upregulated by E2 in presence of ERβ only (14). Grober et al. also observed that several of the same cell growth promoting genes (incl. *MYC, XBP1, MATK* and *FGF18*) to be upregulated by E2 stimulation in wild type MCF7 cells and reduced upon addition of ERβ (although they could not asses if ERβ alone could regulate these genes) and that *JAK2* were E2-upregulated only in presence of ERβ (16). In conclusion, we observe notable differences of key gene regulations that can explain the differently regulated cell proliferative function by respective receptor.

Functional enrichment analysis also supported other differential functions by ERα or ERβ, such as related to cell morphology, cell movement, cell death and survival, several which are consistent with previous studies (9, 16). Previous studies in different cell models have reported that ERβ reduces cell migration (56–59). In our study, liganded ERβ-E2 did indeed repress migration, however, we also found that ERβ homodimers in absence of ligand enhanced cell migration compared to cells with ERα homodimers. A large proportion of the uniquely ERβ-regulated genes also had a cis-regulatory chromatin site that was bound only by ERβ. Upon detailing the binding activity of some of these (*ANXA9* and *TFAP2C)* using ERβ ChIP-qPCR in ERβ (only) and ERα/ERβ (co-expressing) MCF7 cells, we corroborated the ERβ chromatin binding to these sites. These two genes have functions in migration, and their regulations may explain some of the migratory function of ERβ.

Notably, we here also characterized the fraction of genes that were regulated by both ERα and ERβ homodimers, and these were primarily in the same direction. Only very few genes (9 identified) were regulated in opposite directions by the two receptors. We also explored estrogen-regulation of *PKIB, pS2, IL20*, and *GREB1* in greater detail. Three of these were upregulated by both ERα-E2 and ERβ-E2 *(pS2, GREB1*, *PKIB)*. The upregulation of *pS2* by ERβ-E2 supports our previous finding using siRNA of ERα in MCF7- ERβ cells (14). Interestingly, *IL20* gene was upregulated by ERα-E2 but clearly repressed by ERβ-E2 (clone #38), supporting our previous study where we observed that co-expression of ERβ reversed ERα-mediated stimulation of *IL20* (23). This downregulation could however not be replicated in clone #8, where IL20 levels were nearly absent. The genes identified to be commonly regulated by both ERα and ERβ could in theory also include any GPER1 or non-ER dependent E2-mediated signaling that may occur. GPER1 is relatively highly expressed in MCF7 cells, and although it is not a transcription factor, the outcome of its signaling could still impact gene expression. qPCR analysis of some genes in the no-ER cells did not reveal any regulation (**Supplementary Figure 1C**), but this may still be a relevant concern for some commonly regulated genes that lacked an ER-binding site (138 genes, **Figure 5C**). Further, ERα isoforms generated by alternative splicing of the C-leader sequence (lacking exons 1 and 2) may not be deleted following our CRISPR-deletion strategy. The HC-20 antibody is epitope mapped to the C-terminus and have previously been demonstrated to recognize ERα46 (60). Using this antibody **Figure 1B** and **Supplementary Figure 1A**) we note a band the size of ERα46 (between size markers 37 and 50 kDa and below the ERα (66kDa) band). Following CRISPR deletion of exon 1, this band is weaker but still detectable. Thus, we cannot exclude that low levels of ERα46 are still present following knockout. However, we did not detect any effect on target genes (e.g., pS2) in the no ER cells, and the levels are very low in comparison to ERβ and are thus not likely to significantly impact results. A limitation of this study is that only one cell type (MCF7) is analyzed and that we compare cells without Tet (ERβ only) with cells in presence of Tet (ERα only). However, we performed several control experiments, including functional analyses, where we assessed that ERα (only) MCF7 cells responded as both parental (ERα-expressing) and Tet-off mock (ERα-expressing) cells, and our findings of ERα are overall in line with published literature of parental MCF7 cells. We further corroborate ERβ functions in two different MCF7 clones (#8 and #32). Single clones are known to exhibit clonal differences and we note ERβ-increased migration of different levels (faster in #8 compared to #32 under full serum conditions, and lower during low-serum non-estrogenic conditions), some changes in basal gene expression levels (incl. a notable difference in expression of IL20, which is nearly absent in clone #8), and different magnitudes of E2 regulations.

In summary, we here describe the establishment of an estrogen-sensitive cell model which contains ERα homodimer (only) or ERβ homodimer (only) in the same cellular background, providing a novel way to compare the mechanism of ERα and ERβ independently. Our study generates original information on the gene regulatory function of ERβ homodimers in absence of and in response to E2. Some main findings include that ERα or ERβ is essential for MCF7 basal cell growth, that ERβ ligand-independent functions differ from its ligand-dependent functions, such as that ERβ in a ligand-independent manner enhances migration while it reduces migration in response to ligand, and the comprehensively characterization of the estrogen-responsive transcriptional regulation of endogenous genes by the ERβ homodimer. We report that ERβ can modulate unique estrogen-responsive gene profiles that is different from ERα. Our results confirm that the two ERs have opposite effects on cell proliferation, impact cell migration differently, and regulate distinct sets of target genes in response to E2. In conclusion, our findings correlate well with previous studies of ERβ, but reveals distinct transcriptome regulations and demonstrates that ERβ homodimers have both ligand-independent and ligand-dependent functional effects, which can go in different directions.

## Data Availability Statement

The datasets presented in this study can be found in online repositories. The names of the repository/repositories and accession number(s) can be found below: (https://www.ncbi.nlm.nih.gov/, GSE182431).

## Author Contributions

HH, DS, LS, RI, and ZH performed experiments; IS, DS, RI, and HH analyzed data; HH, CZ and CW interpreted results of experiments; DS and HH prepared figures; DS and HH drafted manuscript; DS, L-AH and CW edited and revised manuscript; all authors approved final version of manuscript; CZ and CW initiated and designed the study and CZ supervised HH; CW, CZ and L-AH co-supervised DS, and CW supervised LS and RI. All authors contributed to the article and approved the submitted version.

## Funding

This work was supported by scholarship from the China Scholarship Council (DD and HH), PhD student grants (KID 2-3591/2014 for RI, 2-3707/2013 for HH, 2021-00501 for LS) from the Karolinska Institutet, and funding from the Swedish Cancer Society (21 1632 Pj), the Swedish Research Council (2017-01658) and Region Stockholm (RS2021-0316).

## Conflict of Interest

The authors declare that the research was conducted in the absence of any commercial or financial relationships that could be construed as a potential conflict of interest.

## Publisher’s Note

All claims expressed in this article are solely those of the authors and do not necessarily represent those of their affiliated organizations, or those of the publisher, the editors and the reviewers. Any product that may be evaluated in this article, or claim that may be made by its manufacturer, is not guaranteed or endorsed by the publisher.
