# Supplementary material for: ERα and ERβ Homodimers in the Same Cellular Context Regulate Distinct Transcriptomes and Functions
Source: Front Endocrinol (Lausanne). 2022 Jul 6;13:930227. doi: 10.3389/fendo.2022.930227 (PMC9299245; doi:10.3389/fendo.2022.930227)
Supplement: Supplementary file 1 [file DataSheet_1.docx]

Supplementary Material

# Supplementary Figures


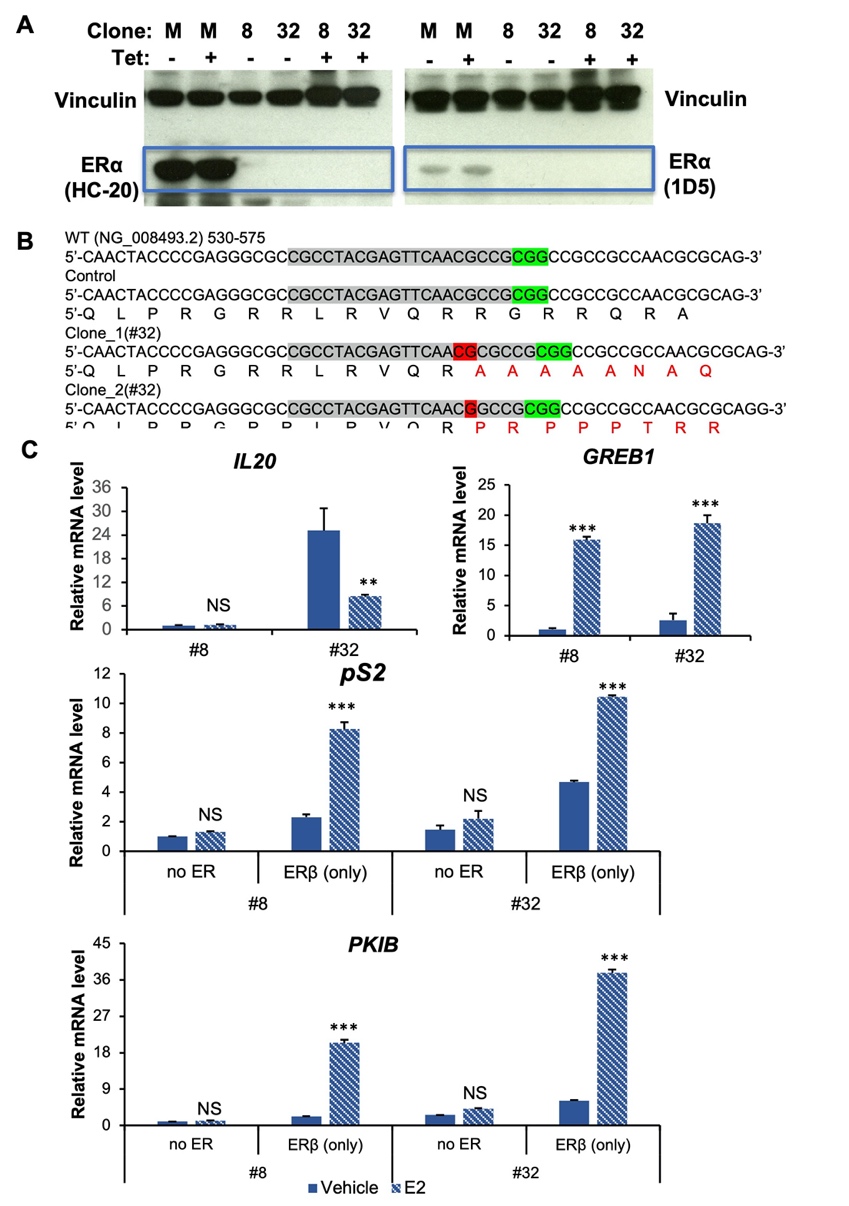


**Supplementary Figure 1**. **The validation of ERα knockout.** (**A**) ERα expression in mock controls and following CRISPR-deletion (clone #8 and #32) with two different ERα antibodies and loading control (Vinculin) Box indicate full length protein (66 kDa). (**B**) Confirmation of gene editing was achieved by PCR amplification of genomic DNA spanning the gRNA target site followed by subcloning and Sanger sequencing. Sequence alignment of bases 530–575 of exon 1 of the RefSeqGene human ESR1 sequence (NG_008493.2). Sequences corresponding to the 20-nt target (grey), 3-nt protospacer adjacent motif (PAM) (green), inserted nucleotides (red), and corresponding amino acids alterations (red) are highlighted. (**C**) mRNA levels of *IL20*, *GREB1, pS2,* and *PKIB* per qPCR in clones #8 and #32 (ERβ-only), with the two lower panels illustrating lack of E2-induced regulations in corresponding no-ER cells. Illustrated as means ± SD from three independent experiments, analyzed by two-way ANOVA followed by Bonferroni test. ***P < 0.001, **P < 0.01, *P < 0.05, NS: not significant

**
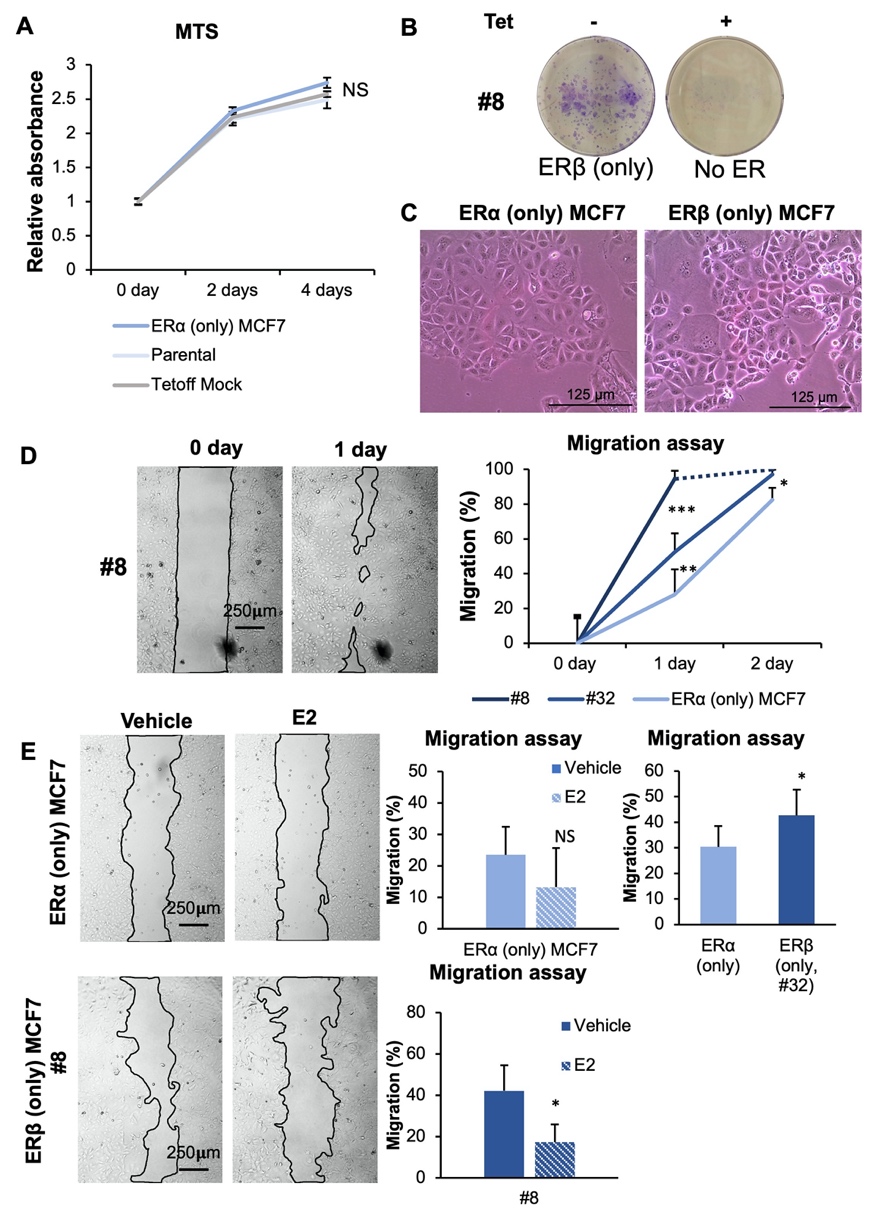
**

**Supplementary Figure 2**. **Proliferative and migratory phenotype of ERα (only) or ERβ (only) MCF7 cells.** (**A**) Cell proliferation assay of ERα (only) parental and Tet-off Mock MCF7 cells. Cells were grown in full-serum medium and measured at day 0, 2 and 4. Absorbance of day 0 was used for normalization. (**B**) Clonogenic assay for ERβ (only) and no-ER cells (clone #8). The cells were cultured with full-serum medium with/without Tet treatment for 8 days. (**C**) Morphology of ERα (only) or ERβ (only, #32) MCF7 cells. (**D-E**) Scratch assay. Cells were seeded into Culture-Insert 4 Well µ-Dish and D) ERα (only), ERβ (only) #8 and #32 were cultured in full-serum medium. ERβ (only) cells (#8 clone) illustrated in left panel. The pictures were taken at day 0, 1 and 2 days after the inserts were removed. Area after migration was measured with Image J and the initial area at day 0 was used for normalization for each cell line (right panel); (**E**) ERα (only) or ERβ (only, #8) MCF7 cells were cultured in low serum (non-estrogenic) conditions for 72h, inserts were removed, and cells were treated with E2 or vehicle. Left panel: Pictures illustrate Day 1 for ERα (only) and Day 2 for or ERβ (only, #8), and (middle panel) corresponding quantification illustrated as means ± SD from three independent experiments. Right panel: ERα (only) or ERβ (only, #32) was compared under vehicle conditions at 24h. A, D was analysed by two-way ANOVA followed by Bonferroni test, E was analyzed by Student’s t-test. ***P < 0.001, **P < 0.01, *P < 0.05, NS: not significant.


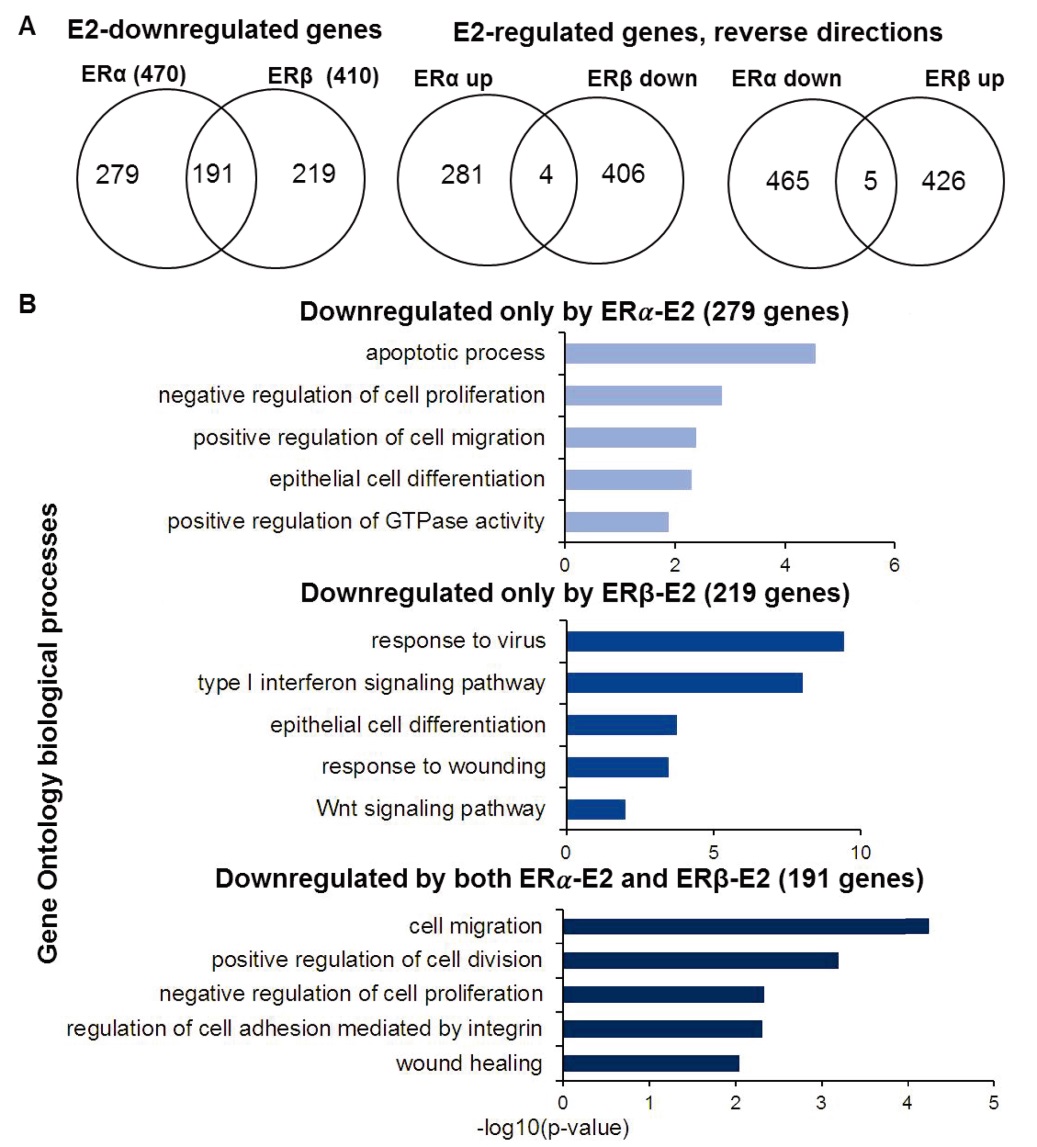


**Supplementary Figure 3. Identification of ERα- and ERβ-specific transcriptome.** (**A**) Comparison of E2-downregulated (left) or genes regulated by ERα or ERβ in opposite manner (middle, right). (**B**) Enrichment analysis of biological functions related to the downregulated gene groups (left A panel) for ERα (279 genes, top) and ERβ (219 genes, middle), and both receptors (191 genes, lower panel) using DAVID.


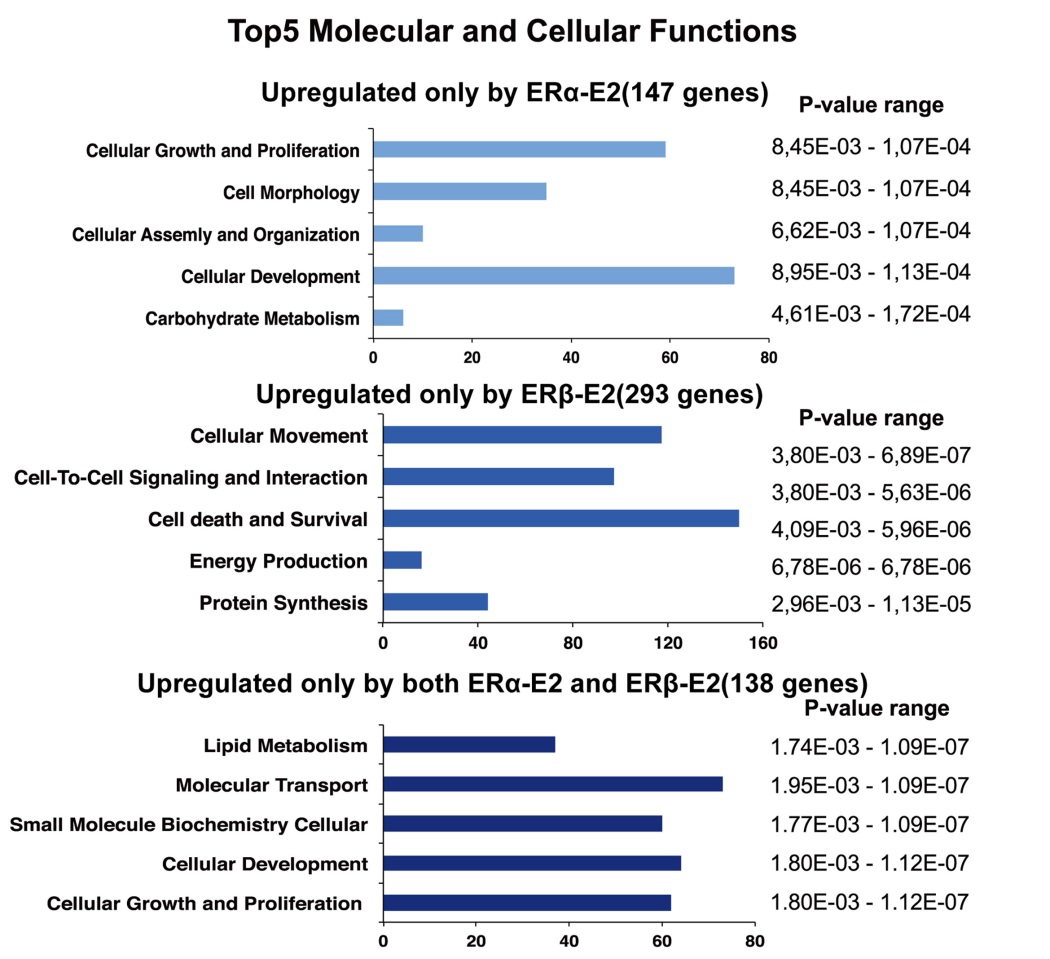


**Supplementary Figure 4. Identification of ERα and ERβ-specific transcriptomes.** Top 5 molecular and cellular functions for the E2-upregulated genes mediated by ERα, ERβ or both (from Fig 4B, lower panel) using Ingenuity Pathway Analysis.


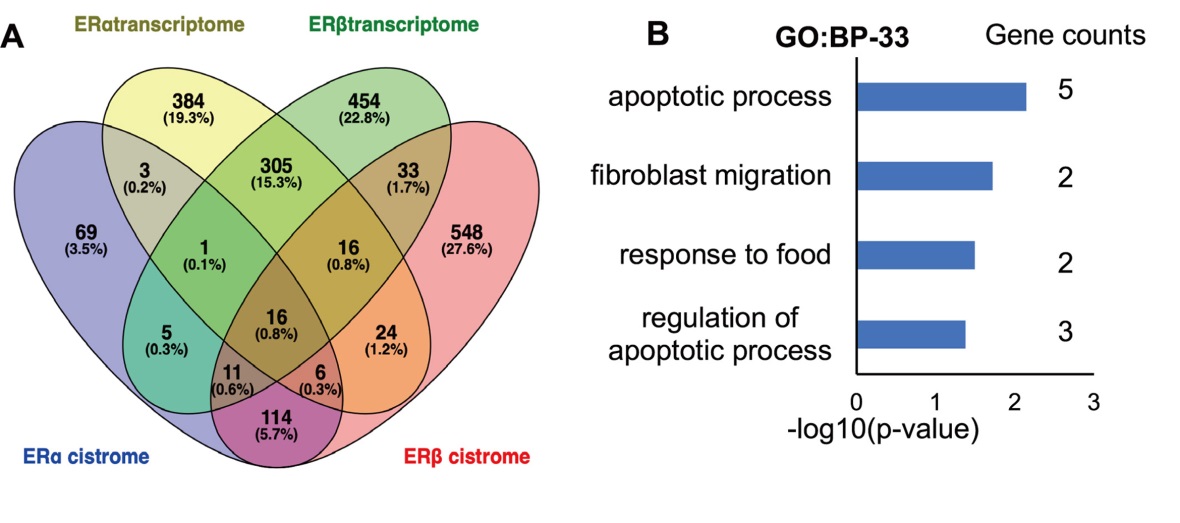


**Supplementary Figure 5. The cistrome and transcriptome data of ERα and ERβ.** (**A**) Venn diagram comparing ERα and ERβ MCF7 transcriptome and cistrome promoter only data. **(B)** GO:BP analysis for 33 uniquely ERβ-regulated genes (transcriptome and promoter cistrome).
